# Impairment effect of infantile coloration on face discrimination in chimpanzees

**DOI:** 10.1098/rsos.211421

**Published:** 2021-11-10

**Authors:** Yuri Kawaguchi, Koyo Nakamura, Masaki Tomonaga, Ikuma Adachi

**Affiliations:** ^1^ Messerli Research Institute, University of Veterinary Medicine Vienna, Vienna, Austria; ^2^ Japan Society for the Promotion of Science (JSPS), Chiyoda-ku, Tokyo, Japan; ^3^ Primate Research Institute, Kyoto University, Kyoto, Japan; ^4^ Faculty of Psychology, Department of Cognition, Emotion, and Methods in Psychology, University of Vienna, Vienna, Austria; ^5^ Faculty of Science and Engineering, Waseda University, Tokyo, Japan; ^6^ Inuyama, Japan

**Keywords:** infantile coloration, chimpanzees, face colour, face shape, face recognition

## Abstract

Impaired face recognition for certain face categories, such as faces of other species or other age class faces, is known in both humans and non-human primates. A previous study found that it is more difficult for chimpanzees to differentiate infant faces than adult faces. Infant faces of chimpanzees differ from adult faces in shape and colour, but the latter is especially a salient cue for chimpanzees. Therefore, impaired face differentiation of infant faces may be due to a specific colour. In the present study, we investigated which feature of infant faces has a greater effect on face identification difficulty. Adult chimpanzees were tested using a matching-to-sample task with four types of face stimuli whose shape and colour were manipulated as either infant or adult one independently. Chimpanzees' discrimination performance decreased as they matched faces with infant coloration, regardless of the shape. This study is the first to demonstrate the impairment effect of infantile coloration on face recognition in non-human primates, suggesting that the face recognition strategies of humans and chimpanzees overlap as both species show proficient face recognition for certain face colours.

## 1. Introduction

Humans extract various sociodemographic information from faces, including gender, ethnicity and age, with high accuracy [1,2]. Face processing in humans is tuned to a specific type of face during their lifetime. For example, adults and older infants can only identify the faces of their own species but not those of other species, while younger infants can equally identify both of them [3]. A number of studies have tested this phenomenon, which is called ‘own-species bias’ [4]. Another example is that humans show better performance in processing faces of their cohort than those of other age classes [5–8]. Thus, adults can identify the faces of adults better than those of children and vice versa [6]. Similar to this own-age bias, efficient face processing is observed for own-race faces compared with other-race faces [9–11]. This superior face processing for certain face types is considered to arise because of the greater exposure of the perceiver to specific face types [4,6,12].

In non-human primates, the advantages of processing certain face types are also observed. For example, captive chimpanzees (*Pan troglodytes*) with less exposure to humans show advantages in face discrimination of own species, while those with lifelong experience with humans perform better face discrimination for humans than for their own species [13]. This result indicates that the amount of experience to certain face types affects the face processing efficiency. In addition, a previous study found that adult chimpanzees show worse face recognition performance towards infant faces than towards adult faces [14]. In this study, chimpanzees were required to match faces of both infants and adults, in an identical matching-to-sample task. The results showed that the response time was longer in discriminating among infant faces than among adult faces, indicating that adult chimpanzees were better at discriminating adult faces than infant faces. However, the mechanisms underlying this impaired facial processing for infant faces are unclear.

Facial shape and colour serve as important cues for extracting various sociodemographic information [2]. Using the morphing technique, researchers have investigated how each facial feature, facial shape and facial colour independently contribute to the perception of various social traits on faces in humans [15–17] and non-human primates [18,19]. Moreover, human face perceptions are attuned to familiar facial shapes and/or colours and altering them to unfamiliar ones distorts efficient face recognition. Some studies have investigated the relative contribution of facial shape and colour to own-race bias [20–22]. They found that not facial shape or colour alone, but both are responsible for poor performance in recognizing other-race faces.

Primate age information can be defined by both facial shape and colour features, at least in some species. A set of morphological features of infants, especially faces (e.g. relatively bigger eyes and forehead, small nose and mouth), which is observed in various species is called ‘baby schema’ [23]. By contrast, of approximately half of all primate species [24], infants have unique skin and/or coat coloration that differs from that of adults [24–27]. Indeed, statistical image quantification has demonstrated that chimpanzee infants also have both unique facial shape and colour characteristics [19]. Chimpanzee infant faces have some baby-schematic characteristics such as bigger eyes located lower in faces and curved supraorbital torus, which contrasts with the straight ones in adults. Moreover, infant faces have pale skin coloration, whereas adult faces have dark skin. An eye-tracking study revealed that chimpanzees' spontaneous attention is attracted to conspecific infant faces, especially infantile facial coloration [28]. In another study, chimpanzees were trained to discriminate between infant and adult faces and tested the generalization of the age category discrimination to morphed face stimuli with facial shape and colour manipulated independently. The results demonstrate that infantile coloration serves as a more robust cue for age category discrimination than a facial shape cue in chimpanzees [19]. Given this evidence, the impaired discrimination performance for infant faces could be caused especially by infantile face coloration. In other words, salient and unfamiliar face features may distract the fluent face processing by chimpanzees by grabbing their attention.

The aim of the present study was to test the impairment effect of infantile face coloration. As illustrated above, chimpanzee infant faces are significantly different from adult faces in both facial shape and colour dimensions [19]. Therefore, it is theoretically possible that discrimination difficulty for infant faces is induced solely by facial shape or colour or by a combination of both. Applying the morphing technique used in a previous study [19], we created four types of face stimuli by independently manipulating shape and colour: (i) faces with adult shape and adult colour, (ii) faces with adult shape and infant colour, (iii) faces with infant shape and adult colour, and (iv) faces with infant shape and infant colour. We compared face identification performance in a face-matching task across four stimulus conditions and tested which facial feature, shape or colour, had the greater effect on the difficulty of face identification. Since a previous study found that chimpanzees have a higher sensitivity to face colour than shape cues related to age difference [19], we predicted that specific face colour, rather than specific face shape, would make infant face recognition difficult in chimpanzees.

## 2. Material and methods

### 2.1. Participants

Six adult chimpanzees participated in the experiment (five females and one male, aged 18–42 years). All of them had experiences of interacting with conspecific infants and two of them had previously reared their offspring (table 1). The participants voluntarily came to the experimental booth (1.8 × 2.15 × 1.75 m) and joined the experiment. During the experiment, they were not restrained and could quit the experiment whenever they wanted. The chimpanzees lived socially at the Primate Research Institute, Kyoto University. All chimpanzees had previously participated in the study which used morphed stimuli of infant and adult faces [19] and the other study showing superior face processing for adult faces over infant faces [14]. The chimpanzees lived in an enriched environment, which featured both indoor and outdoor enclosures, and had free access to water and received food (fresh fruits, vegetables, sweet potatoes and nutritionally balanced biscuits) several times each day.

**Table 1.** Participant information.

individual name (GAIN^a^ ID)	sex	age	birth experience
Ai (0434)	female	43	parous
Ayumu (0608)	male	20	—
Chloe (0441)	female	39	parous
Cleo (0609)	female	20	nulliparous
Pal (0611)	female	19	nulliparous
Pendesa (0095)	female	43	nulliparous

^a^GAIN (the Great Ape Information Network) is the information network about Hominoidea living in Japan.

### 2.2. Apparatus

The experiment was conducted in an experimental booth (1.8 × 2.15 × 1.75 m). A matching-to-sample task was conducted using a 17-inch touch-sensitive LCD monitor (IO Data LCD-AD172F2-T, 1280 × 1024 pixels) and a universal feeder delivered food reward (Biomedica, BFU310-P100). Stimulus presentation, response detection and feedback were controlled by a customized program written in Microsoft Visual Basic 2010 Express working on a personal computer (CPU: Core™ i3-4130 3.40 GHz; Intel, Santa Clara, CA, USA).

### 2.3. Stimuli

**Figure 1.**
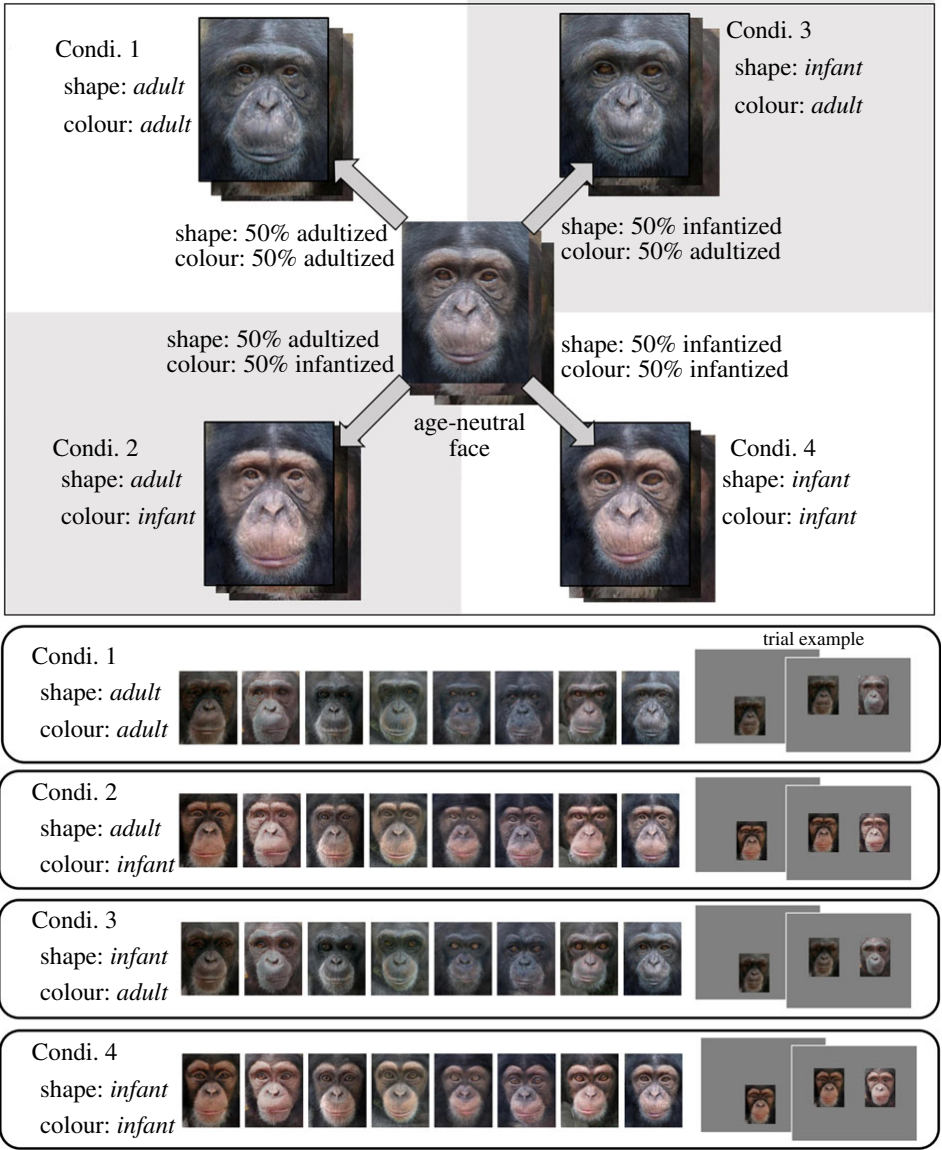
Morphed stimuli. Sample stimuli and comparison stimuli were constantly assigned from the same condition.

Eight adult and eight infant faces (four female and four male faces for each), which were used in a previous study [19], were delineated by 118 landmarks as described previously [19]. The original images depicted faces of chimpanzee infants aged 2–10 months and adults aged 13–22 years at the time the pictures were taken. We confirmed that both facial shape and colour equally effectively characterize the difference between infant and adult faces by analysing these original faces; we conducted a principal component analysis (PCA) for facial shape and colour and found that PC1 of both shape and colour equally well contribute to the differences between infant and adult faces (electronic supplementary material). To make a set of ‘age-neutral’ faces, one infant face and one adult face chosen at random were superimposed, resulting in eight age-neutral faces (figure 1). Each original image was used only once. Then, the age-neutral images were transformed into either 50% adultized or 50% infantized for a dimension of facial morphology (hereafter referred to as ‘shape’) and a dimension of skin reflectance including both face colour and texture (hereafter referred to as ‘colour’). This resulted in four types of morphed stimuli: (i) faces with adultized shape and adultized colour, (ii) faces with adultized shape and infantized colour, (iii) faces with infantized shape and adultized colour, and (iv) faces with infantized shape and infantized colour (figure 1). For stimuli creation, we used Webmorph (v. v0.0.0.9001) [29], an online program for face transformation with algorithms based on previous human face studies [15,30]. We prepared eight stimuli for each condition and none of them was familiar to the participants. Importantly, the eight faces had some physical variation within each condition, but the extent of the variation among them was the same among the four conditions and only the baseline shape and colour differed depending on the condition.

### 2.4. Procedures

**Figure 2.**
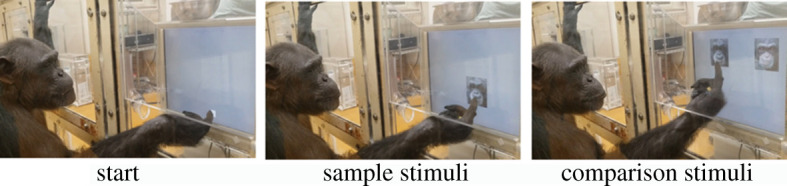
Matching-to-sample task.

We conducted an identical matching-to-sample task (figure 2). In the task, each trial started immediately after the participant touched the start key. The start key appeared twice: horizontally in different places at the bottom of the monitor and the second was always presented at the centre. Once the participant touched the start key, a sample stimulus appeared at the centre of the monitor for 500 ms. Then, it disappeared and two comparison images were presented. One of the comparison images was identical to the sample stimulus and the other was the face with different identity selected from the same shape and colour manipulation condition. The participants were required to touch an image identical to the sample. When they chose the correct answer, a food reward (a piece of apple) and a chime sound were delivered. When they chose the wrong answer, no food reward was delivered and a different sound was played. During a session, the trial order of the conditions and stimuli was pseudo-randomized. There were 56 combinations of the two images. For each combination, there were two arrangements (left or right), resulting in 112 unique trials for each of the four conditions. A total of 448 unique trials were divided into eight sessions, which were repeated four times. One session consisted of 56 trials and two sessions were completed each day.

### 2.5. Analysis

The accuracy and response time were analysed using a generalized linear mixed model (GLMM) to test whether the shape and colour of the stimuli significantly affect the face recognition performance. All the analyses were conducted with R 3.5.1 (R Core Team, 2018) using the ‘lme4’ package [31]. For accuracy, the answer to each trial (correct versus incorrect, coded 1 or 0, respectively) was included as a response variable, and the shape of the stimuli (adultized or infantized), the colour of the stimuli (adultized or infantized) and their interaction were included as explanatory variables. We used a binomial distribution for accuracy. We included participants as random intercepts and random slopes for all fixed effects and session numbers as random intercepts. To determine the best model, we compared five models, namely, a model including the interaction between shape and colour (full model), a model with both main effects of shape and colour without the interaction, a model with only a main effect of colour, a model with only a main effect of shape and a null model with only intercept. We selected the best model based on the Akaike information criterion (AIC). When the difference between the lowest AIC model and other candidate models is less than 2 AIC units, we chose the simpler model. In addition, the response time was analysed in the same way as the accuracy, except that the gamma distribution was applied. Only data from the correct trials were analysed. Based on [32], the response times that exceeded the mean + 3 s.d. for each participant were excluded from the analysis, resulting in 0.6% of the data being discarded.

## 3. Results

**Figure 3.**
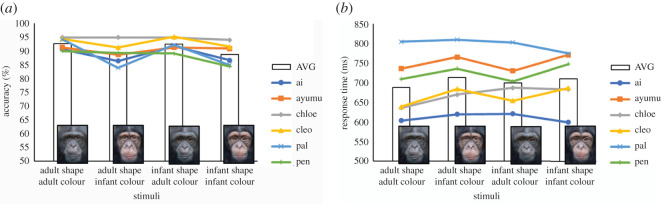
Accuracy (*a*) and response time (*b*).

Figure 3 shows the mean accuracy and mean response time for each condition. For accuracy data, the model with only a main effect of colour was selected as the best model because of the minimum AIC (AIC = 6573.0; table 2). The result of this model showed a significant main effect of colour (*β* = −0.41, s.e. = 0.12, *z* = −3.53, *p* < 0.01, 95% confidence interval (CI) [−0.64, −0.18]). On average, face identification was disrupted by 3.6% more in the infant colour condition than in the adult colour condition. For the response time data, the AIC of the model with the main effect of both shape and colour (−4236.7) and the model with only a main effect of colour (−4235.7) is almost equivalent (i.e. differ by less than 2 AIC units); therefore, we selected a simpler model, which is the model with only a main effect of colour. Although the model with main effect of colour was selected as the best model, the result of this model did not show a significant main effect of colour (*β* = 0.03, s.e. = 0.01, *t* = 1.37, *p* = 0.08, CI [−0.00, 0.06]). The response time was increased by 18 ms in the infant colour condition compared with the adult colour condition, on average. Visual inspection of the demographic factors such as the sex or birth experience of the participants did not reveal any systematic individual differences.

**Table 2.** The fit of each GLMM model. Italics indicates the selected models.

model	AIC	BIC	log likelihood	deviance
accuracy				
colour × shape	6589.4	6698.6	−3279.7	6559.4
colour *+* shape	6580.3	6653.1	−3280.1	6560.3
* colour*	*6573.0*	*6616.7*	*−3280.5*	*6561.0*
shape	6619.3	6663.0	−3303.6	6607.3
null	6613.9	6635.7	−3303.9	6607.9
response time				
colour × shape	−4234.5	−4119.7	2133.3	−4266.5
colour + shape	−4236.7	−4157.8	2129.4	−4258.7
* colour*	*−4235.7*	*−4185.5*	*2124.9*	*−4249.7*
shape	−4210.4	−4160.2	2112.2	−4224.4
null	−4208.2	−4179.5	2108.1	−4216.2

## 4. Discussion

The present study investigated the impairment effect of infantile coloration on face discrimination in chimpanzees using a well-controlled face morphing technique. As predicted, the results showed that chimpanzees made more mistakes when matching faces with infant coloration, regardless of the face shape. The response time was also slightly longer for faces with infant coloration, but the difference was not significant. These results indicate that infant facial colour, rather than facial shape, impairs face recognition in chimpanzees. The recognition of infant faces in chimpanzees is improved by altering the facial colour into that of adults. This study is the first to demonstrate the interference effect of specific face coloration on face recognition in non-human primates. These results are consistent with previous findings on the role of facial coloration in the age perception of the faces of chimpanzees [19].

Why do chimpanzees show worse performance for faces with infantile coloration? One of the explanations of human own-race and own-age bias is based on the amount of visual experience with faces. That is, frequent exposure to a certain type of face results in the acquisition of *face schema*, a face prototype, and faces that deviate from this schema are processed less efficiently [12]. In this study, our chimpanzees had experience interacting with infants, but had not been exposed to them for a while when they were tested. Moreover, not only for our chimpanzee participants but also for adult chimpanzees in general, social interaction and visual experience with infants probably occur less frequently than with adults. Thus, less exposed infant faces are probably outside the face tuning and not as well processed as usual faces with the expertise-based strategy of chimpanzees.

The difference in the amount of experience between adult and infant faces by itself does not fully explain why the performance of the chimpanzees was decreased by infant face colour but not shape. First, it should be noted that the different performances among the conditions were not due to low-level visual confounds of the facial stimuli. Rather, face variations among stimuli remain constant among the four conditions due to the controlled way of creating the stimuli. Therefore, if chimpanzees had matched faces based solely on physical differences between the given two stimuli, their performances would not differ among the conditions. Moreover, image analysis of adult and infant faces has demonstrated that both shape and colour are independently and equally effective in characterizing the difference between adult and infant faces. In this study, we manipulated face texture as well when we manipulated face colour of the stimuli. Thus, it is theoretically possible that infant facial texture also affected the performance although visual inspection does not find the robust difference of the texture between infant and adult faces. Previous studies found that infantile face coloration especially attracts the attention of chimpanzees [19,28]. For chimpanzee perceivers, infant faces are characterized by facial colour instead of shape, and their fluent face recognition may be disrupted by the unfamiliar and salient colour. Once chimpanzees categorize an infant face as an atypical face, which may be easily done by its coloration, more detailed usual face processing may not continue in a similar manner to a ‘usual’ adult face. Some previous human studies have investigated the relative contribution of facial colour and shape to the own-race bias by using morphed stimuli such as ours [20–22]. Interestingly, in contrast with our results with chimpanzees, these previous human studies found that not solely facial shape or facial colour, but both are responsible for poor performance in recognizing other-race faces. Of course, it would be too simplified to directly compare impaired recognition of infant faces in chimpanzees and the own-race bias in humans. Nevertheless, this contrast is intriguing because it may suggest that humans and chimpanzees have different face recognition strategies.

The potential function of infantile colour has been discussed for decades, and some hypotheses have been proposed, including the alloparental hypothesis [25,26], infant defence hypothesis [24] and paternity cloak hypothesis [24]. The current results do not contradict the paternity cloak hypothesis, which assumes that infant coloration makes identity and paternity detection difficult. It was proposed that the unique natal coat colour might prevent males from identifying phenotypic marker signalling paternity based on physical appearance, leading to reduced infanticidal risk. We found that infantile coloration makes chimpanzees' face identification less accurate. However, the current study was not designed to test the paternity cloak hypothesis. Thus, it remains unclear whether particular visual cues, which are important for kin recognition, are also masked by the coloration. Moreover, testing the adaptive function of the infantile face colour during evolution is challenging. Nevertheless, it may be possible that infantile face colour makes the identity and potential paternity of chimpanzee infants difficult to detect in the wild. In this regard, the face colour variations among species or subspecies of the genus *Pan* are worth noting. Infantile facial coloration exists in chimpanzees, but not (or much less conspicuously) in bonobos (*Pan paniscus*) [33]. Moreover, the conspicuousness of infantile coloration varies even among subspecies of chimpanzees [33]. In the present study, both the participants and the individuals depicted in the original images of the stimuli were western chimpanzees (*Pan troglodytes verus*). Future studies should test species with more conspicuous and less conspicuous infantile coloration (i.e. eastern chimpanzees (*Pan troglodytes schweinfurthii*) and bonobos) as both participants and stimuli.

There are some limitations and remaining questions that future studies should consider. First, it remains to be seen how specific or general our finding is: whether the colour is limited to infant face coloration, but not other coloration and whether the context is limited to conspecific face recognition but not the recognition of other types of stimuli. Related to this point, testing whether other species (e.g. humans) also show similar results would also be informative. If the impaired effect of infantile coloration on face recognition in chimpanzees results from the asymmetric amounts of experience between adult and infant faces, humans with less experience of chimpanzees will not show the same effect. However, chimpanzee experts who have regular experiences with adult chimpanzees will show similar results to chimpanzees. Nonetheless, it should be noted that the novelty of the stimulus is unlikely to explain these results. If novelty, the lack of experience, simply affects the results, chimpanzees would have shown worse performance for the face stimuli with infant shape and adult colour, and for the face with adult shape and infant colour because participants have never seen such ‘incongruent’ faces before. However, there was no interaction effect between face shape and colour on matching difficulty. Although some questions remain unanswered, this study is the first to demonstrate the impaired effect of certain face colours on discriminability. The present study shows that the face recognition strategies of humans and chimpanzees overlap as both species show proficient face recognition for certain face colours, although humans but not chimpanzees show proficient face recognition for certain face shapes.

## Data accessibility

The data are provided in electronic supplementary material [34].
